# The Role of Ethnic Prejudice in the Modulation of Cradling Lateralization

**DOI:** 10.1007/s10919-020-00346-y

**Published:** 2020-10-27

**Authors:** Gianluca Malatesta, Daniele Marzoli, Luca Morelli, Monica Pivetti, Luca Tommasi

**Affiliations:** Department of Psychological, Health and Territorial Sciences, University “G. d’Annunzio” of Chieti-Pescara, Blocco A Psicologia, Via dei Vestini, 31, 66100 Chieti, Italy

**Keywords:** Left-cradling bias, Hemispheric specialization, Blatant racism, Subtle racism, Social cognition, Mother-infant relationship

## Abstract

The left-cradling bias is the tendency to cradle an infant on the left side, regardless of the individuals’ handedness, culture or ethnicity. Many studies revealed associations between socio-emotional variables and the left-side bias, suggesting that this asymmetry might be considered as a proxy of the emotional attunement between the cradling and the cradled individuals. In this study we examined whether adult females with high levels of prejudice toward a specific ethnic group would show reduced left-cradling preferences when required to cradle an infant-like doll with ethnical features of the prejudiced group. We manipulated the ethnicity of the cradled individual by asking 336 Caucasian women to cradle a White or a Black doll and then assessed their prejudice levels toward African individuals. Significant correlations were shown only in the Black doll group indicating that the more the prejudice toward Africans, the more the cradling-side preferences shifted toward the right. Furthermore, participants exhibiting low levels—but not those exhibiting high levels—of ethnic prejudice showed a significant left-cradling bias. These findings show that ethnic prejudice toward the specific ethnic group of the cradled individual can interfere with the left preference in the cradling woman. The present study corroborates our suggestion that the left-cradling bias might be considered as a natural index of a positive socio-communicative relationship between the cradling and cradled individuals. On the contrary, the right-cradling bias might be considered as a cue of the presence of affective dysfunctions in the relationship.

## Introduction

With the term “left-cradling bias” we refer to the lateral preference in holding/cradling—or even imagining holding/cradling—an infant with his/her head to the left of the cradling individual’s body midline for non-feeding purposes. Cradling behavior is usually considered as a subset of behaviors in which an infant is held close to the body, and it more precisely refers to a holding position in which the infant is kept in a supine posture between one’s arm and trunk. This is reflected in the studies that have appeared on the topic since the first scientific publication by Lee Salk in 1960, although in many of them a more vertical posture of the infant (i.e., against one’s shoulder, with the arm flexed to protect and secure her/him) was also included in such an operational definition.

Research showed that both women (over 65%) and men (albeit to a weaker degree) exhibit a left-side preference in cradling behavior (for reviews, see Donnot and Vauclair 2005; Packheiser et al. 2019b). Although it is generally considered a maternal predisposition, the left-cradling bias has also been observed in nulliparous women and young girls handling dolls (de Château and Andersson 1976; Forrester et al. 2019). Additionally, it has been shown that, when the cradling individual sooths/interacts with the infant/doll without being engaged in a “functional” cradling interaction (i.e., with a specific purpose such as feeding the infant or inserting a pacifier into the mouth of the infant/doll, according to the definition of van der Meer and Husby 2006) essentially with feeding purposes, the left-side preference is independent of handedness (e.g., Donnot 2007; van der Meer and Husby 2006; see Packheiser et al. 2019b for a review). Although many explanations have been proposed for this phenomenon (for a review, see Harris 2010), it is increasingly accepted that the left-cradling bias stems from an evolutionarily wired preference in both mothers—and, perhaps, infants—to choose a positioning fostering the communication of socio-emotional information via their right cerebral hemisphere in both humans and other mammals (Giljov et al. 2018; Manning and Chamberlain 1991; Sieratzki and Woll 2002). In this regard, the right hemisphere of the human brain—and therefore the part of environment falling within the individual’s left hemispace—is predominantly specialized for the processing of visual, auditory, chemical, and haptic socio-emotional stimuli (Brancucci et al. 2009). Interestingly, some studies found that left-cradling individuals showed a stronger leftward asymmetry for the processing of emotions from faces (e.g., Bourne and Todd 2004; Harris et al. 2001; Harris et al. 2010). If the left-cradling bias seems to reveal the asymmetrical brain organization typically shown by mothers in infant-monitoring functions on the one hand, on the other hand, it might foster a typical neurodevelopment in the cradled infants by exposing them for longer to a flow of information during one of the most important critical periods in their life (Hendriks et al. 2011). For example, a link has been suggested between the left-sided positioning received during infanthood and a typical neurodevelopment later in life (Jones 2014; Malatesta et al. 2020a, c), including the development of a right-lateralized network for the perception of the human face (Vervloed et al. 2011), a hemispheric asymmetry that seems to be patent especially for female faces (Parente and Tommasi 2008; Prete et al. 2017). Very recently, a new chapter has been added to the history of the biobehavioral cradling system. In fact, it has been suggested that (from the mother’s point of view) the left-cradling bias might not only place the infant in the cradler’s left visual hemifield, but also expose their left profile, namely that containing the left hemiface (Malatesta et al. 2020b), which is the one displaying the greater expressiveness and readability (Hendriks et al. 2011).

However, in light of the conspicuous literature on human cradling, reducing the complexity of this lateralized behavior to a mere issue of motor or perceptual asymmetries seems to be simplistic. For example, the role of hemispheric specialization for face processing in modulating the laterality of cradling in women has not always been confirmed (e.g., Donnot and Vauclair 2007; Harris et al. 2019; Lucas et al. 1993), and even when it has, the effect size observed is rather small, suggesting that other factors are involved. Moreover, much cradling research has focused on the emotional state of the cradler. Based on the groundbreaking research conducted by Salk (1960, 1973), who first resurrected the scientific debate on such a leftward asymmetry (also showing a reversed lateral pattern as a result of postnatal separation between mother and infant), many investigations have been conducted to demonstrate the existence of a relationship between atypical (i.e., right) lateral preferences on the one hand and either dysfunctions in the affective state of the cradling individuals or aberrations in the emotional connection between the cradling and the cradled individuals on the other hand. Specifically, it was shown that the presence of depression (Malatesta et al. 2019b; Pileggi et al. 2020; Scola et al. 2013; Weatherill et al. 2004), anxiety (Vauclair and Scola 2009), stress (Reissland et al. 2009), social pressure (Boulinguez-Ambroise et al. 2020), non-secure attachment with one’s own mother and romantic partner (Malatesta et al. 2019a), and—possibly as a consequence of anxiety, stress and/or depression—postpartum separation (Salk 1973) may somehow reduce the occurrence of the left-cradling bias in women. It is plausible that the typical (i.e., left) cradling patterns of lateralization can be reversed by a disruption of the cradler’s affective and mental state, which might influence the emotional connection between the cradling and the cradled individuals and thus entail perturbed interactions. In this regard, de Château et al. (1978) found that right-cradling mothers showed reduced body contact with their babies during the neonatal period compared with left-cradling mothers. Moreover, reduced or absent socio-communicative abilities seem to be reflected in a shift from left to right in cradling asymmetry. For example, recent research showed that the left-sided pattern is related to inherent empathic and social competencies (Fleva and Khan 2015; Forrester et al. 2019; Malatesta et al. 2019b; Pileggi et al. 2015).

Many studies specifically aimed at investigating the universality of the left-cradling bias across cultures and ethnicities (Bolton 1978; Brüser 1981; Richards and Finger 1975; Saling et al. 1983; Saling and Cooke 1984; Schiefenhövel 1980), and across centuries of history (Alvarez 1990; Grüsser 1983; Finger 1975; Salk 1973) indicating that such an asymmetry is not affected by socio-cultural factors but is perhaps determined by a set of hereditary factors uniformly spread throughout all human populations. The only known exceptions to this universal rule of left-cradling bias in healthy samples are found in Malagasy (an indigenous population of Madagascar), where most adults, both women and men, seem to hold babies on the right (Nakamichi 1996) and in Tanka (a population living on boats on the banks of the rivers in Southern China), where women feed their infants exclusively from the right breast (Ing et al. 1977), possibly due to the right-sided opening of the women’s traditional dress in this population.

Although this asymmetrical behavior has been analyzed in several ethnic groups by many researchers, it is surprising that no study has ever verified whether cradling an infant of another ethnic group with respect to the cradling woman, would disrupt the left-side preference and whether any socio-emotional variable might be involved therein. In the present study, we aimed to fill this gap in cradling literature. Moreover, given that it has been previously shown that the left-sided positioning is an index of the socio-emotional attunement between the cradling and cradled individuals, we hypothesized that the presence of prejudice toward a different ethnic group in women cradling a doll representing an infant belonging to the prejudiced group might shift the typical left preference toward the atypical right preference. Besides the possible role of ethnic prejudice in modulating the lateral preference in women cradling a different-race doll, we might expect that women would be more likely to cradle on the left a doll representing an infant belonging to their own rather than another ethnic group. In particular, they could respond more positively to the former because in such a condition, they can more easily imagine cradling their own baby, a kin baby, or at least an own-race baby. On the other hand, much research showed that alloparental behaviors are present in humans reflecting an uncontrollable instinct of taking care of vulnerable beings (e.g., Ashdown and Faherty 2020; Bentley and Mace 2009), which could induce positive responses also in the case of a different-race doll.

Prejudice is traditionally considered a natural and common “antipathy based upon a faulty and inflexible generalization” (Allport 1954; p. 9), involving both affective and cognitive components. Based on Allport’s theory, Pettigrew and Meertens (1995; see also Coenders et al. 2001) suggested that prejudiced attitudes form ideological clusters of beliefs useful to justify consequent discrimination, and that such psychological constructs are composed of two factors: blatant prejudice and subtle prejudice. With regards to blatant prejudice, it is characterized by fear of the outgroup and refusal of close contact with outgroup individuals, a sort of anti-intimacy component. The subtle prejudice is a modern and indirect form of prejudice, characterized by the defense of the ingroup’s traditional values, exaggeration of cultural differences, and denial of positive emotional responses toward the outgroup (Arancibia-Martini et al. 2016).

In Europe and the United States, racial prejudice (i.e., holding negative attitudes toward individuals based on their race) of White individuals toward Black people is a pervasive problem (e.g., Albarello and Rubini 2012; Haslam et al. 2002; Orsi et al. 2010; Quillian et al. 2019; Zick et al. 2008). People’s tendency to differentiate White from Black people is so strong that studies have also shown the automatic stereotyping based on racial category and Afrocentric facial features (Blair et al. 2004). High level of blatant and subtle prejudice toward Blacks and Moroccans has been found among Italians. Blatant prejudice was a significant predictor of attitude toward immigration (Manganelli Rattazzi and Volpato 2001; Mancini and Carbone 2007).

As for the relations between racial prejudice and emotions, the Intergroup Emotion Theory (IET; Mackie et al. 2008; Smith and Mackie 2008) argues that “intergroup emotions” arise when people identify with a social group and respond emotionally to events or objects that impinge on the group. Self-categorizing as an ingroup member determines emotional responses, especially for highly identified group members. The emotions one feels when considering Black people, Muslims, gay men, or immigration policies depend on how you are thinking about yourself. Emotions such as anger, fear, disgust, or envy targeted specifically at an outgroup may relate to perceptions, prejudiced attitudes, or discriminatory behaviors directed at the outgroup.

In line with research indicating reduced empathic reactions toward individuals belonging to a prejudiced racial outgroup (e.g., Forgiarini et al. 2011), it is plausible to hypothesize that a situation in which the cradling individual has a prejudice toward the cradled individual is comparable to a situation in which the cradling individual has a general lack of empathy (Fleva and Khan 2015; Malatesta et al. 2019b; Pileggi et al. 2015). However, given that a significant left-cradling bias comparable to that of females has been found in males (e.g., Harris et al. 2019), but to a lesser extent (with some exceptions for new fathers; Dagenbach et al 1988; Harris et al. 2007; Scola and Vauclair 2010), any factor modulating such a side preference should be more likely to emerge in women. Therefore, in this study, we examined whether women with high levels of prejudice toward a specific ethnic group would show reduced left-cradling preferences when asked to take in their arms and soothe an infant-like doll with ethnic features of the prejudiced group compared to women with low levels of prejudice and/or those asked to cradle an infant-like doll with ethnic features of their own group.

## Method

### Participants

Three hundred and thirty-six Italian White women took part in the experiment. Given that they were randomly recruited in the campuses of the University of Chieti and Pescara (Italy) and among the experimenters’ (four female and two male psychology students) acquaintances, most participants were university students. Their age ranged from 18 to 54 years (M = 25.68 ± 0.38), and 23 of them were not right-handers (i.e., scored zero or negatively on the Italian version of the Edinburgh Handedness Inventory; Salmaso and Longoni 1985). Given that handedness might represent a confounding factor in studies relating cradling lateral preferences to other psychological traits (e.g., a reduced left-cradling bias has been reported in left-handers; e.g., Huheey 1977; van der Meer and Husby 2006; see Packheiser et al. 2019b for a meta-analysis), we excluded non-right-handed participants. Therefore, the examined sample consisted of 313 right-handed (M = 82.99 ± 0.95) women (age range 19–54 years; M = 25.78 ± 0.4), of which 156 were included in the “same-race doll” group (see below; age range 18–50 years; M = 26.08 ± 0.54) and 157 in the “different-race doll” group (see below; 19–54 years; M = 25.48 ± 0.59). Participants were not required to provide information about their country of origin, marital status, and parity. All participants gave written informed consent to participate in the study by signing an authorization form. Neither invasive nor risky procedures were involved, and the data were analyzed anonymously. The study was conducted in accordance with the principles of the Declaration of Helsinki. All procedures followed the guidelines of the Italian Association of Psychology Ethical Code and of the local ethical committee.

### Procedure and Materials

**Fig. 1:**
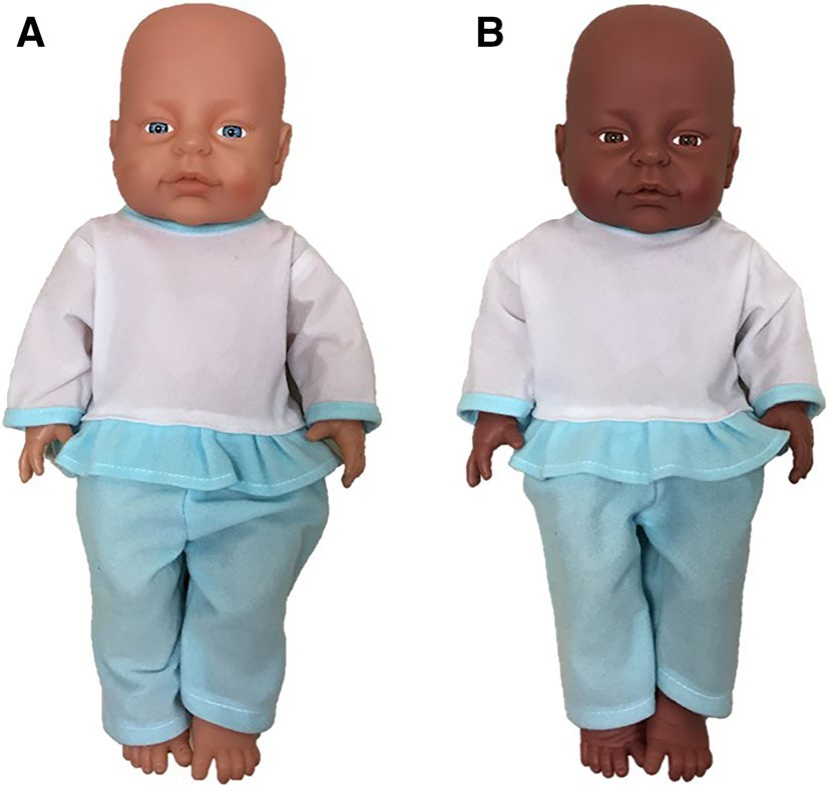
The same-race doll (**a**) and the different-race doll (**b**) used in the study

As regards the assessment of participants’ cradling-side preferences, they were randomly divided into two equal-number cradling-task groups: 168 participants were required to perform the cradling task using a same-race doll (White), and 168 participants were required to perform the cradling task using a different-race doll (Black; Fig. 1).

*Cradling task*. The assessment of the “cradling-bias index” was the same as in a previous study carried out by Malatesta et al. (2019a). Participants were led by the experimenter to a quiet room in which they performed the cradling task. The experimenter, positioned behind an empty table in front of the participant, informed her that she would perform a series of trials in which she had to pick up a life-like doll having the approximate size (45-cm length) and appearance of a baby positioned on the table. In the “same-race doll” group, participants were required to cradle a doll displaying some Caucasian-like ethnic features (fair skin and light eyes; Fig. 1a); in the “different-race doll” group, participants were required to cradle a doll displaying some Black African-like ethnic features (dark skin and brown eyes; Fig. 1b). Except for the ethnic features, the two dolls were identical in weight, size, and clothing (a white and blue baby dress).

**Fig. 2:**
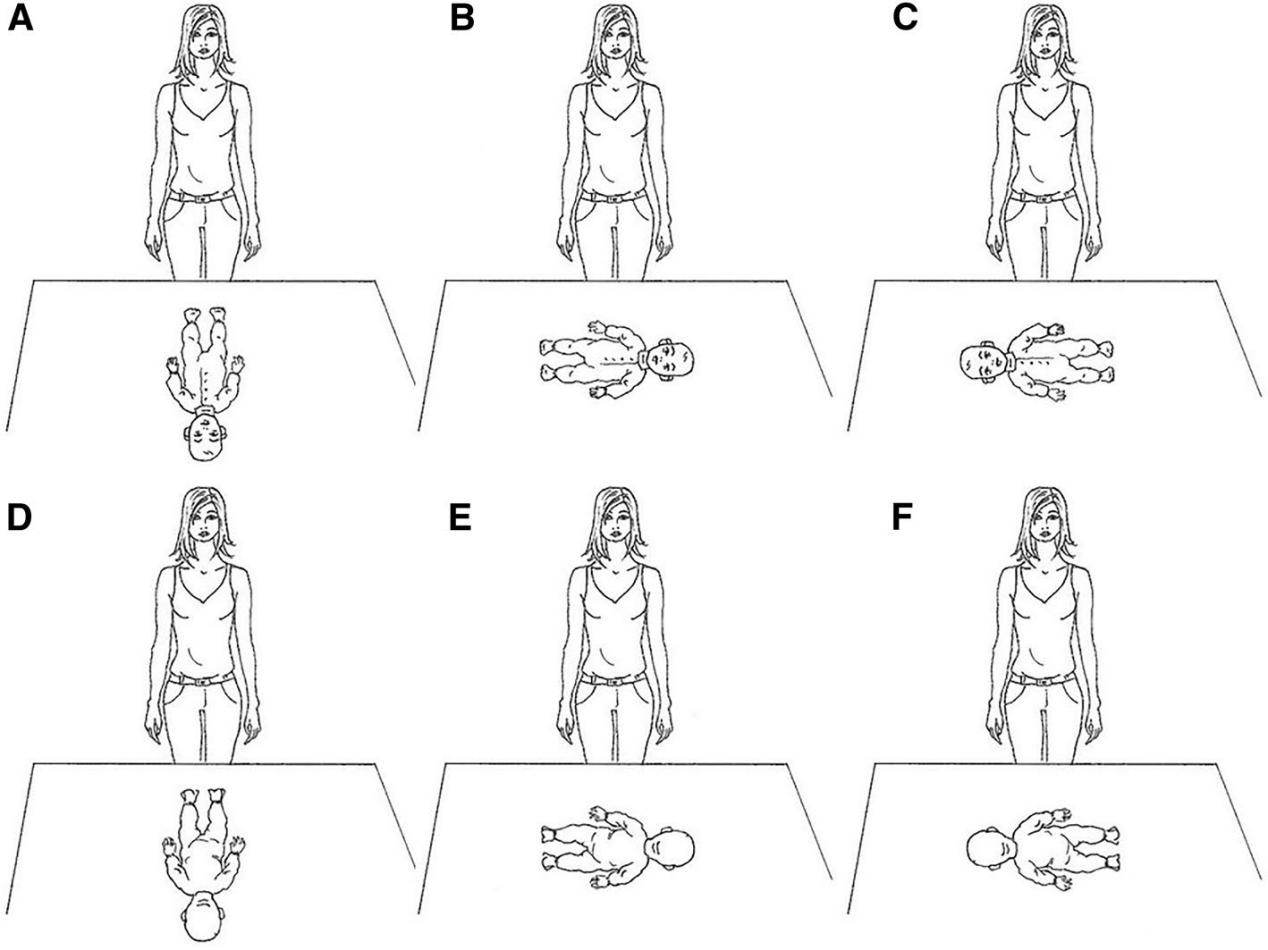
Graphic representation of the six trials performed by each participant in the cradling task

The participant performed six trials, in each of which the same question was asked: “Imagine that this doll is a real infant who is crying: please take it in your arms and soothe it”. Only in the other-race experimental group, the experimenter stressed that it was an “African” infant. After the participant had held the doll for about 8–10 s, the experimenter said: “Thank you, you can put it back on the table”. For each trial, the experimenter positioned the doll opposite to the participant, laying it in one of six different positions (whose order was counterbalanced across subjects): supine with the head on the center with respect to the participant (Fig. 2a), supine with the head on the left (Fig. 2b), supine with the head on the right (Fig. 2c), prone with the head on the center (Fig. 2d), prone with the head on the left (Fig. 2e), and prone with the head on the right (Fig. 2f). Participants could see the experimenter placing the doll between consecutive trials, but none of them asked about the role of doll positioning for the task. According to the head positioning of the doll chosen on each occasion (regardless of whether a more horizontal or a more vertical holding was performed), the experimenter coded each trial in which the participant cradled to the left as − 1, each trial in which the participant cradled to the right as +1, and each trial in which the participant cradled to the midline as 0. Therefore, the “cradling-bias index” ranged from − 6 (indicating an absolute left-cradler) to +6 (absolute right-cradler), with scores of zero representing no cradling-side bias at all (unbiased cradlers) because of the lack of a clear-cut cradling preference.

Participants scoring negatively (i.e., from − 1 to − 6) on the cradling-bias index were labeled as “left-cradlers” and those who scored positively (i.e., from +1 to +6) were labeled as “right-cradlers”. After the cradling task, participants were required to fill in the following survey.

*Blatant and subtle prejudice scale*. This is a self-reported scale for measuring two types of intergroup prejudices toward a target out-group: blatant and subtle (Pettigrew and Meertens 1995; Italian version by Arcuri and Boca 1996). Blatant prejudice is a traditional form of ethnic racism involving a physical rejection and an emotional resistance against any contact with the out-group. Subtle prejudice is, by contrast, less target-directed than blatant prejudice, and is expressed in a more acceptable way in Western cultures. Pettigrew and Meertens (1995) identified three components of subtle prejudice: the defense of traditional values, the exaggeration of cultural differences, and the denial of positive emotions. The scale consists of 20 items: 10 items measure blatant prejudice (e.g., “Most Black people living here who receive support from welfare could get along without it if they tried”) and 10 items measure subtle prejudice (e.g., “Black people living here should not push themselves where they are not wanted”). The target out-group we used was African immigrants. As of 2019 data, 11,471 immigrants disembarked on the Italian coasts. Starting from 2014 to 2019, between 20% and 50% of immigrants come originally from North and Central African countries such as Algeria, Nigeria, Ivory Coast, Senegal, Mali, and Sudan. The number of unaccompanied children dropped from approximately 13,000 in 2014 to 1700 in 2019 (Source: Fondazione ISMU on data of the Ministry of the Interior). In medium-size cities of Southern/Central Italy (such as the ones where data were collected) the presence of immigrants is very low as most of them reside in bigger cities in Northern Italy, where they can reconnect with their community of origin and have better job opportunities. Participants indicated their agreement or disagreement concerning the content of each item on a 5-point Likert scale with scores calculated by adding the items (for each scale, scores ranged from 0 to 50, with a conventional cut-off separating high and low scores between 30 and 31; Pettigrew and Meertens 1995). High scores on the scales indicate high levels of prejudice. On the basis of the scores obtained in each scale, Pettigrew and Meertens (1995) identified four categories of individuals: “equalitarians”, who score low on both scales; “subtles”, who score low on the blatant scale and high on the subtle scale; “bigots”, who score high on both scales; “type 0”, who score high on the blatant scale and low on the subtle scale.

## Results

### Comparisons Between Groups

As shown in Table 1, no statistical differences were observed between the “same-race doll” and the “different-race doll” groups regarding the variables of interest, as well as age and laterality quotient.

**Table 1:** Differences between the “same-race doll” and the “different-race doll” groups according to participants’ age, laterality quotient, cradling-bias index, blatant and subtle prejudice score means [in square brackets the standard errors]

Variable	Same-race	Different-race	*t*(*df*)	*p*
Age	26.08 [0.54]	25.48 [0.59]	*t*(311) = 0.752	.453
Laterality quotient	89.98 [1.31]	88.46 [1.39]	*t*(311) = 0.794	.428
Cradling-bias index	− 0.59 [0.26]	− 0.92 [0.27]	*t*(311) = 0.887	.376
Blatant prejudice score	19.84 [0.45]	20.34 [0.48]	*t*(311) = − 0.754	.452
Subtle prejudice score	28.23 [0.45]	28.22 [0.41]	*t*(311) = 0.023	.981

### Cradling-bias Index

Participants showed a significant left-cradling bias against chance in both the “same-race doll” group (*M* = − 0.59 [54.91%]; *t*(155) = − 2.269; *p* = .025) and the “different-race doll” group (*M* = − 0.92 [57.7%]; *t*(155) = − 3.394; *p* = .001).

### Correlations between Cradling-bias Index and Prejudice Scores

**Fig. 3:**
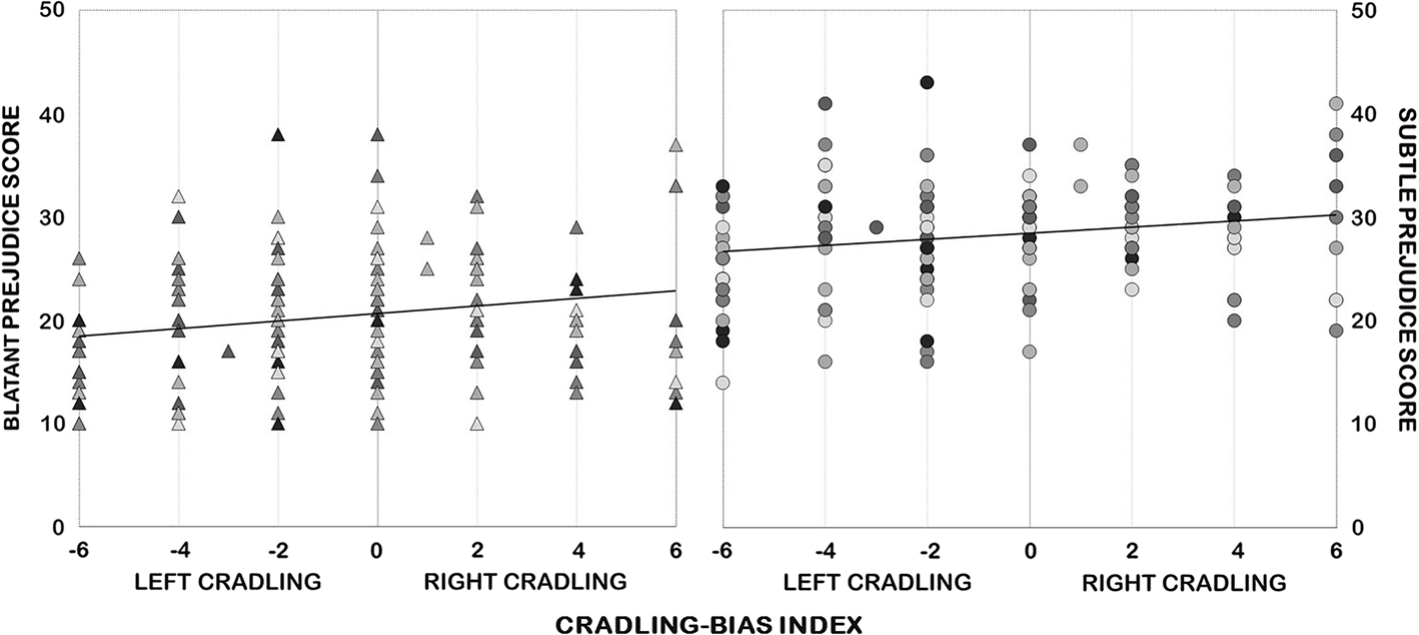
Scatterplots of the cradling-bias index and blatant (left panel) and subtle (right panel) prejudice scores for participants in the “different-race doll” group

As regards the “same-race doll” group, data analysis did not show significant correlations between the cradling-bias index and both the blatant (*r*(156) = .032; *p* = .692) and the subtle (*r*(156) = -.036; *p* = .654) prejudice scores. On the contrary, in the “different-race doll” group, participants’ cradling-bias index showed a significant positive correlation with the blatant (*r*(157) = .207; Bonferroni-corrected *p* = .037) prejudice score and an almost significant positive correlation with the subtle (*r*(157) = .195; Bonferroni-corrected *p* = .057) prejudice score (see Fig. 3), indicating that the higher the prejudice toward Africans, the more the right-cradling preferences.

### Cradling-bias Categories

Participants scoring negatively (from − 1 to − 6) on the cradling-bias index were labelled as left-cradlers, those scoring zero as unbiased cradlers, and those scoring positively (from +1 to +6) as right-cradlers. For the “same-race doll” group, different proportions of left-cradlers (*n* = 79 [50.6%]), unbiased cradlers (*n* = 27 [17.3%]), and right-cradlers (*n* = 50 [32.1%]) were observed (χ^2^(2) = 26.115; *p* < .001). Specifically, a significantly larger proportion of participants were categorized as left-cradlers rather than unbiased cradlers (χ^2^(1) = 25.509; *p* < .001) and right-cradlers (χ^2^(1) = 6.519; *p* = .011), and a significantly larger proportion of participants were categorized as right-cradlers rather than unbiased cradlers (χ^2^(1) = 6.870; *p* = .009). Furthermore, for the “different-race doll” group, different proportions of left-cradlers (*n* = 81 [51.6%]), unbiased cradlers (n = 31 [19.7%]), and right-cradlers (*n* = 45 [28.7%]) were observed (χ^2^(2) = 25.427; *p* < .001). Specifically, a significantly larger proportion of participants were categorized as left-cradlers rather than unbiased cradlers (χ^2^(1) = 22.321; *p* < .001) and right-cradlers (χ^2^(1) = 10.286; *p* = .001), and no difference was observed between the proportions of participants categorized as right-cradlers and unbiased cradlers (χ^2^(1) = 2.579; *p* = .108).

Given that: i) only 58 (27 of which belonging to the “same-race doll” group and 31 to the “different-race doll” group) out of 313 participants were categorized as unbiased cradlers, ii) it would have been problematic to further split these small subsamples according to the various prejudice categories, and iii) we were specifically interested in the relationship between prejudice and the laterality (left vs right) of cradling, we excluded from further analyses such participants because of their limited numerosity and lack of a clear-cut cradling preference.

### Prejudice Categories

With regards to participants’ classification according to the prejudice categories, 169 (87 of which belonging to the “same-race doll” group and 82 to the “different-race doll” group) participants were labeled as “equalitarians”, 74 (37 of which belonging to the “same-race doll” group and 37 to the “different-race doll” group) as “subtles”, 11 (4 of which belonging to the “same-race doll” group and 7 to the “different-race doll” group) as “bigots”, and 1 (belonging to the “same-race doll” group) as “type 0”.

Given the relatively small numbers of participants falling into several categories, in order to perform the data analysis all categories different from equalitarians were collapsed. Therefore, for each prejudice measure we compared participants labelled as “equalitarians” with those labelled as “non-equalitarians”. Our sample was distributed as described in Table 2.

**Table 2:** Sample distribution for prejudice categories according to Pettigrew and Meertens (1995)

	Equalitarians	Non-Equalitarians			
		Subtles	Bigots	Type 0	Total
Same-race doll group	87	37	4	1	42
Different-race doll group	82	37	7	–	44

**Fig. 4:**
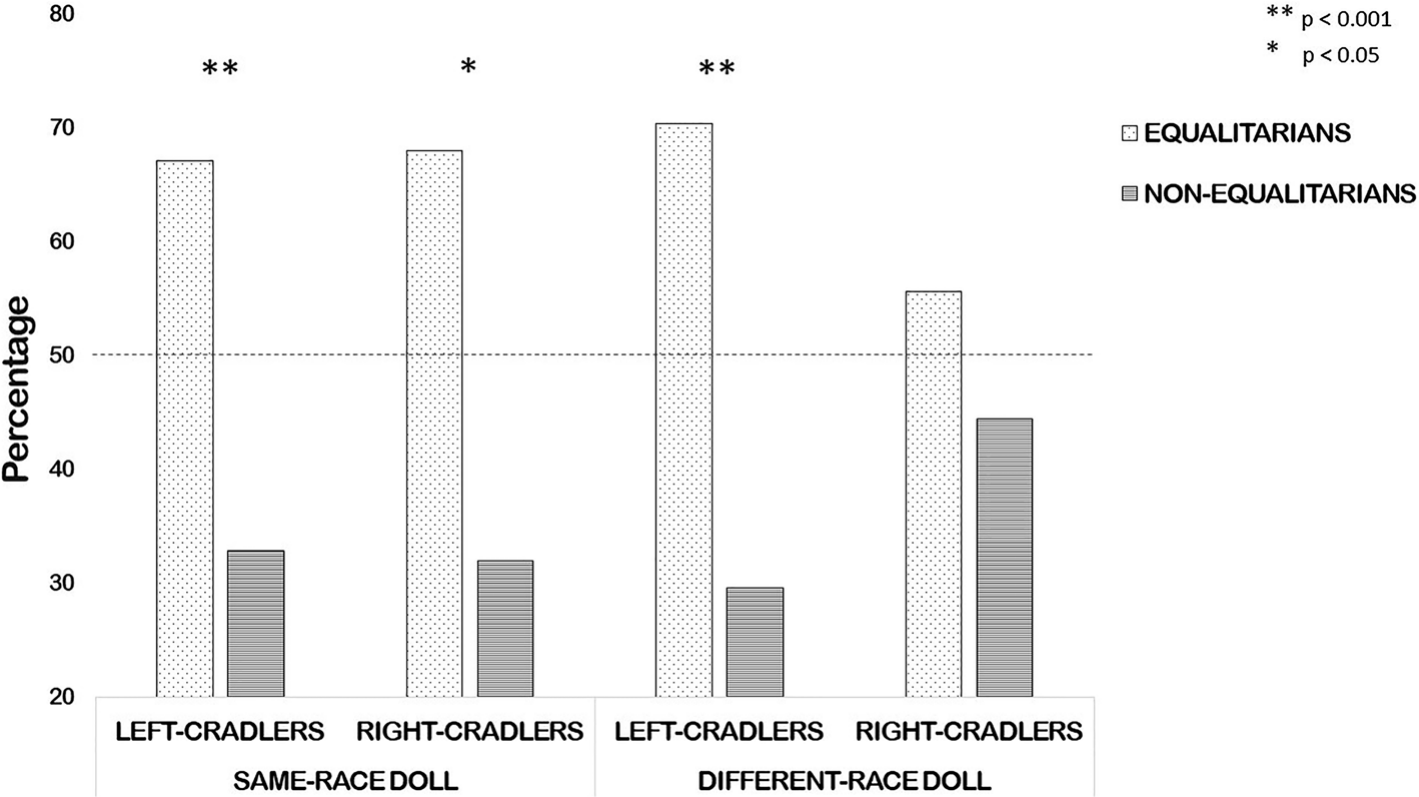
Percentage of equalitarians and non-equalitarians according to cradling categories (left-cradlers or right-cradlers) and experimental condition (“same-race doll” or “different-race doll” group). Note: the dashed line indicates the chance level [50%]

*Cradling-side bias and prejudice categories.* With regards to the “same-race doll” group, left-cradlers were significantly more likely to be equalitarians (*n* = 53 [67.1%]) rather than non-equalitarians (*n* = 26 [32.9%]; χ^2^(1) = 9.228; *p* = .001), and right-cradlers showed a similar difference between the proportions of equalitarians (n = 34 [68%]) and non-equalitarians (*n* = 16 [32%]; χ^2^(1) = 6.48; *p* = .044). As regards the “different-race doll” group, left-cradlers were significantly more likely to be equalitarians (*n* = 57 [70.4%]) rather than non-equalitarians (*n* = 24 [29.6%]; χ^2^(1) = 13.444; *p* < .001), but right-cradlers did not show any significant difference between the proportions of equalitarians (*n* = 25 [55.6%]) and non-equalitarians (*n* = 20 [44.4%]; χ^2^(1) = 1.140; *p* = .456; Bonferroni correction was applied to adjust for multiple comparisons; Fig. 4).

## Discussion

In this study, we investigated for the first time whether a lateral preference exists when women cradle a doll of another ethnic group rather than a doll of their own ethnic group. So far, many studies proved significant left-cradling bias in mothers and nulliparous women across nations, continents and history (e.g., Alvarez 1990; Saling et al. 1983; Saling and Cooke 1984), but none of them examined whether the same side preferences held true when the cradling and the cradled individuals belonged to different ethnic groups. To this aim, we tested a large sample of women in a laboratory setting using a life-like doll, a method that has been largely used in literature (see Donnot and Vauclair 2005 for a review) and that is capable of assessing the cradling-side preference with reasonable reliability and validity compared to real infants (whose differences in age, size, weight, along with uncontrolled motoric and postural variables, might somehow interfere with a reliable assessment of the lateral preference; for a meta-analysis, see Packheiser et al. 2019b).

Rather unexpectedly, our results did not show any significant difference in the proportion of left, unbiased, and right-cradlers between the two groups: participants requested to take the different-race doll in their arms exhibited a significant left-cradling bias comparable to that exhibited by participants requested to take the same-race doll in their arms. According to this result, the manipulation of the ethnic features of an infant-like doll cannot induce—alone—an atypical lateral pattern in cradling behavior of women. In other words, there is no difference in lateral preferences when cradling an infant belonging to one’s own or another ethnic group. Therefore, it is possible to broadly infer that, for White women, cradling a same- or different-race infant can implicitly elicit caregiving behaviors similar to those evoked by infant facial features in both mothers (Thompson-Booth et al. 2014) and nulliparous women (Glocker et al. 2009a, b; Cárdenas et al. 2013). In this regard, both behavioral (Proverbio et al. 2011) and electrophysiological (Proverbio and De Gabriele 2019) studies showed that this attentional bias toward infant faces—rather than adult faces—also overlooked the well-known own-race bias (i.e., the tendency to better recognize and categorize same- rather than other-race faces; Malpass and Kravitz 1969). Although the hemispheric specialization in cradling behavior has been widely investigated, in particular regarding the associations with the left-visual field bias for the processing of emotions from faces (e.g., Bourne and Todd 2004; Harris et al. 2001, 2010), there are conflicting opinions about the cerebral lateralization pattern of the own-race bias for faces. If, on one side, the own-race bias in face perception has been related to greater activity in the right (likely due to its greater involvement in holistic processing; Correll et al. 2011; Davis et al. 2016; Hellige et al. 2010) rather than left hemisphere, on the other side, a recent report showed a left-hemispheric dominance for ethnic group categorization (Prete and Tommasi 2018), possibly indicating a featural processing (left hemisphere) of own-race faces in contrast to a holistic processing of other-race faces (right hemisphere; see Hugenberg et al. 2010). In this complicated framework, our study lies in the middle, with no effect of ethnic congruency on cradling-side preferences, which turned out to be left-side biased regardless of whether the doll displayed the cradling woman’s ethnical features or otherwise. Future studies are needed to explicitly or implicitly assess the participants’ own-race bias by using categorization tasks in association with the cradling task used in the present study.

However, when data were analyzed according to participants’ prejudice scores, interesting associations were found. First, significant correlations between cradling side preferences and prejudice scores were observed in participants required to cradle a different-race doll, but not in those required to cradle a same-race doll. Specifically, increases in both blatant and subtle prejudice scores were associated with reduced left-cradling preferences. As no differences were observed in the “same-race doll” group, we propose that women prejudiced toward Africans are more likely to show a rightward asymmetry in cradling behavior when required to take a (Black) African doll (and—presumably—infant) in their arms.

Such an interpretation is also confirmed when frequency analyses were conducted according to the cradling and prejudice categories in which participants were labelled. In fact, we found a significantly larger proportion of equalitarians (scoring lower in both the prejudice scales) rather than non-equalitarians (scoring higher in at least one of the two scales of prejudice, or in both) in both left- and right-cradlers for the “same-race doll” group, and in left-cradlers for the “different-race doll” group. More interestingly, no significant difference was shown in the proportion of equalitarians and non-equalitarians in right-cradlers of the different-race group. Namely, equalitarians represented the significant majority of the sample in both the control and experimental groups, but this aspect ratio was reduced to a non-significant difference only in right-cradling women required to cradle an African doll.

According to the present data, the laterality of cradling exhibited by a woman when handling a different-race doll/infant seems to be associated, to some extent, with the prejudice of that woman toward individuals belonging to such an ethnic group. Instead, prejudice levels per se cannot be associated with the laterality of cradling. Some possible considerations can be provided for the present pattern of results. The fact that individuals with high levels of prejudice tend to refuse contact with (or rather unconsciously deny positive emotional responses to) out-group individuals (Cottrell and Neuberg 2005; Pettigrew and Meertens 1995) could explain why, when the out-group target is represented by an out-group infant (or doll) to cradle, such an unconscious motivation to avoid emotional attunement might interfere in typical dyad dynamics, with a relative shift of cradling-side preferences from the (typical) left to the (atypical) right side. As observed in past research, the right-cradling bias is associated with post-partum separation (Salk 1960, 1973) and less body contact between mother and infant, especially in the early neonatal interactions (de Château et al. 1978). Additionally, right-cradling mothers revealed fewer feelings of affinity, and were more detached from and less responsive to their infants’ communicative feedback compared to left-cradling mothers (Bogren 1984; de Château et al. 1982; Turnbull and Lucas 2000). Similarly, maternal depression, which is related to a reduction of left-side preferences in infant-holding (Malatesta et al. 2019b; Pileggi et al. 2020; Weatherill et al. 2004), might elicit similar mechanisms as those involved in prejudice, leading women to experience less positive emotional interactions when cradling infants. It is possible to speculate that different psychological factors such as depression, lack of empathy, and racial prejudice can result in significant underestimations of the emotional signals coming from the cradled infant and, thus, in an impairment of the relationship (Field 1992). In addition, such results might be discussed in light of recent findings on the lateralization of other instances of social touch (such as kissing and embracing), among which cradling is included. Specifically, individuals exhibit a population-level lateralization (albeit with a leftward rather than rightward asymmetry) in two other instances of social touch (i.e., embracing and kissing). As seen in cradling, these lateralized behaviors are affected by several variables such as social pressure and handedness, as well as by the emotional context (for a review, see Ocklenburg et al. 2018). In particular, it has been shown that a positive or negative emotional context could shift the rightward asymmetry in embracing significantly to the left (Packheiser et al. 2019a, 2020) with a significantly reduced laterality quotient when social touch was conducted in negative emotional situations (Packheiser et al. 2020). In this regard, we could speculate that the “different-race” doll situation might have induced a negative affect in participants prejudiced toward Africans, thus reducing the left-cradling bias.

An alternative explanation of the results lies in the researches on stereotype activation. With respect to racial stereotypes, Devine (1989) proposed that because of common socialization experience, both low and high prejudiced Whites have the same group-based representation of Blacks and automatically activate these negative representations when they encounter a member of that group. When controlled processing is possible, low prejudiced Whites inhibit this tendency, whereas high prejudiced Whites continue to show this effect (Kawakami et al. 1998). We argue that participants cradling a Black doll could activate the anti-Black prejudice, leading to an avoidance or distance-taking behavior with the target doll. This reasoning is based on previous findings by Dovidio et al. (2002), who argue that while Whites have full access to their explicit attitudes and are able to monitor and control their more overt and deliberative behaviors, they do not have such full access to their implicit attitudes or to their less monitorable behaviors. The authors found that explicit attitudes primarily predicted deliberative verbal behaviors while implicit attitudes (as measured with response latency) mainly predicted spontaneous behaviors such as nonverbal friendliness (Fazio 1990; Wilson et al. 2000). Asking participants to cradle a Black doll could have activated the relevant negative stereotype, leading to an avoidance or distance-taking (spontaneous and nonverbal) behavior with the target group. This avoidance behavior was expressed in terms of increased right-cradling. Results showed no correlation between the cradling-bias index and prejudice scores for the “same-race doll” group, as the White doll did not activate any stereotype. In summary, our results seem to corroborate once again the role of the left-cradling bias as a potential index of the emotional attunement between the cradling and the cradled individuals and, more generally, of the emotional connection between the cradling woman and others.

### Limitations of the Current Study

Some limitations of the present study should be considered. First, we should point out that the doll we used for the different-race experimental group displayed only some of the Black African-like ethnic features (dark skin and brown eyes). Therefore, in that condition, the experimenter stressed that it was an “African” infant. Such an emphasis, added only in the experimental group, might have somehow affected participants’ responses both in the cradling task and in the prejudice evaluation. Future studies could fix this issue by using dolls actually showing Caucasian-like and Black African-like (or Asian-like, as well, considering that prejudiced attitudes toward such a group might have been enhanced by the recent COVID-19 pandemic) ethnic features, rather than stressing this before the experimental task. Future studies should also address the influence of the sex of the infant/doll on the lateralization of the cradling behavior, a variable that has often been neglected by investigations on cradling. In fact, although our participants were not required to indicate the perceived sex of the doll, they might have assumed that—regardless of its ethnicity and due to its white and blue baby dress—it represented a male.

Moreover, it should be noticed that some researchers distinguish between different types of infant holding, for example, by contrasting proper cradling (sometimes indicated as arm-holding, in which the infant is horizontally held supine in one’s own arms) with upright holding (sometimes indicated as shoulder-holding, in which the infant is vertically held against one’s own shoulder or trunk; Harris et al. 2019; Todd and Banerjee 2016; Todd and Butterworth 1998; Vauclair and Donnot 2005). Nonetheless, in the present paper we established to use the term cradling in its wider sense (without distinguishing between the different types of hold; e.g., horizontal and vertical holding), also because of the specific task involved (participants were asked to take in their arms and soothe a doll) and because we were interested only in the side, not in the final type of posture assumed. In this regard, it should be remarked that several studies investigating the relationship between cradling-side preferences and several psychological variables did not discriminate between or conflated the different types of hold (e.g., Pileggi et al. 2015; Scola et al. 2013; Weatherill et al. 2004; Reissland et al. 2009).

Finally, the scale used to measure prejudice toward Black people, namely Pettigrew and Meertens’ scale (1995), was developed in Europe, and its use is widespread in this context (Gawronski et al. 2003; Hofmann et al. 2005; van Dick et al. 2004; Vrij et al. 2003). The scale owes its popularity to its having been described as a bidimensional structure, allowing the assessment and comparison of the strengths of the old (i.e., blatant) and new (i.e., subtle) forms of prejudice. Ironically, both forms of prejudice sound “old-fashioned” for the contemporary readers. Moreover, we are aware that the scale has been criticized in terms of factorial structure (Coenders et al. 2001) and discriminant concurrent validity (for the Italian version: Gattino et al. 2008; Leone et al. 2006). However, this scale is still used in recent studies targeting prejudice toward Chinese people and Turkish immigrant (e.g., Tabri et al. 2020; Van Dessel et al. 2020).

## Data Availability

The data that support the findings of this study are included as electronic supplementary materials.
